# 5-HT_2A_ receptor loss does not alter acute fluoxetine-induced anxiety and exhibit sex-dependent regulation of cortical immediate early gene expression

**DOI:** 10.1042/NS20180205

**Published:** 2019-02-01

**Authors:** Minal Jaggar, Toshali Banerjee, Noelia Weisstaub, Jay A. Gingrich, Vidita A. Vaidya

**Affiliations:** 1 Department of Biological Sciences, Tata Institute of Fundamental Research, Homi Bhabha Road, Colaba, Mumbai 400005, India; 2 Department of Physiology, Faculty of Medicine, University of Buenos Aires, UBA-CONICET, Paraguay 2155 3er piso, Buenos Aires C1121ABG, Argentina; 3 Department of Psychiatry, Columbia University, New York, NY 10032, U.S.A.; 4 Division of Developmental Neuroscience, Sackler Institue for Developmental Psychobiology and New York State Psychiatric Institute, New York, NY 10032, U.S.A.

**Keywords:** antidepressant, anxiety, gene expression, prefrontal cortex, serotonin receptor

## Abstract

*Background:* Acute treatment with the selective serotonin reuptake inhibitor (SSRI), fluoxetine (Flx), induces anxiety-like behavioral effects. The serotonin_2A_ receptor (5-HT_2A_) is implicated in the modulation of anxiety-like behavior, however its contribution to the anxiogenic effects of acute Flx remains unclear. Here, we examined the role of the 5-HT_2A_ receptor in the effects of acute Flx on anxiety-like behavior, serum corticosterone levels, neural activation and immediate early gene (IEG) expression in stress-responsive brain regions, using 5-HT_2A_ receptor knockout (5-HT_2A_^−/−^) mice of both sexes. *Methods:* 5-HT_2A_^−/−^ and wild-type (WT) male and female mice received a single administration of Flx or vehicle, and were examined for anxiety-like behavior, serum corticosterone levels, FBJ murine osteosarcoma viral oncogene homolog peptide (c-Fos) positive cell numbers in stress-responsive brain regions of the hypothalamus and prefrontal cortex (PFC), and PFC IEG expression. *Results:* The increased anxiety-like behavior and enhanced corticosterone levels evoked by acute Flx were unaltered in 5-HT_2A_^−/−^ mice of both sexes. 5-HT_2A_^−/−^ female mice exhibited a diminished neural activation in the hypothalamus in response to acute Flx. Further, 5-HT_2A_^−/−^ male, but not female, mice displayed altered baseline expression of several IEGs (brain-derived neurotrophic factor (*Bdnf*), *Egr2, Egr4*, FBJ osteosarcoma gene (*Fos*), FBJ murine osteosarcoma viral oncogene homolog B (*Fosb*), Fos-like antigen 2 (*Fosl2*), Homer scaffolding protein (*Homer*) 1-3 (*Homer1-3*), Jun proto-oncogene (*Jun*)) in the PFC. *Conclusion:* Our results indicate that the increased anxiety and serum corticosterone levels evoked by acute Flx are not influenced by 5-HT_2A_ receptor deficiency. However, the loss of function of the 5-HT_2A_ receptor alters the degree of neural activation of the paraventricular nucleus (PVN) of the hypothalamus in response to acute Flx, and baseline expression of several IEGs in the PFC in a sexually dimorphic manner.

## Introduction

Selective serotonin reuptake inhibitors (SSRIs), including fluoxetine (Flx), are the most commonly prescribed antidepressants given their improved side effect profile compared with other major classes of antidepressants [1]. However, it is important to note that treatment with SSRIs such as Flx is known to exert potent anxiogenic behavioral effects, in particular during the acute phase of treatment, an observation that is also noted in animal models [2,3]. The acute effects of Flx treatment differ from those noted following chronic administration, namely increased anxiety in the acute phase of treatment and a shift to anxiolytic effects observed on sustained treatment [3]. Furthermore, acute Flx is known to evoke enhanced activity of the stress-responsive, hypothalamus–pituitary–adrenocortical (HPA) axis leading to elevated circulating corticosterone levels, whereas sustained Flx administration is reported to normalize HPA axis dysfunction in animal models of depression and in clinical studies [4,5]. The acute phase of Flx treatment through induction of a ‘hyperserotonergic’ state [6] may overlap to a certain degree with the nature of state induced by acute stress, which is known to activate the HPA axis, enhance serotonin release, and induce anxiety-like behavior. The contribution of specific serotonergic receptors to the acute anxiogenic effects of Flx treatment remains at present unclear, though prior studies have implicated the 5-HT_2_ receptor subtypes in contributing to the anxiogenic effects of acute SSRI treatment [7–9].

The G_q/11_-coupled serotonin_2A_ (5-HT_2A_) and serotonin_2C_ (5-HT_2C_)receptor are highly expressed in limbic neurocircuitry, and have been strongly implicated in the regulation of anxiety-like behavior [9–11]. Studies have raised the possibility that the acute anxiogenic effects of SSRIs, may involve a contribution of the 5-HT_2C_ receptor in the bed nucleus of the stria terminalis (BNST) [6] and basolateral nucleus of the amygdala (BLA) [8], with a down-regulation of the 5-HT_2C_ receptor suggested to mediate a shift toward anxiolysis following sustained SSRI administration [12]. Adjunct administration of the 5-HT_2A_ receptor selective antagonist (*R*)-(+)-α-(2,3-Dimethoxyphenyl)-1-[2-(4-fluorophenyl)ethyl]-4-piperinemethanol (MDL100907) along with SSRIs is reported to improve SSRI efficacy on specific behavioral tasks [13]. However, the contribution of the 5-HT_2A_ receptor to the acute anxiogenic effects of SSRIs still remains unclear.

Here, we sought to examine the contribution of the 5-HT_2A_ receptor to the acute effects of the SSRI, Flx, on anxiety-like behavior and circulating corticosterone levels, using 5-HT_2A_ receptor knockout mice (5-HT_2A_^−/−^) [10]. Further, we addressed the degree of neural activation evoked by acute Flx in the paraventricular nucleus (PVN) of the hypothalamus and the prefrontal cortex (PFC) in 5-HT_2A_^−/−^ and WT mice, and gene expression of several activity-regulated, immediate early genes (IEGs) in the PFC. 5-HT_2A_^−/−^ mice have been previously reported to exhibit a baseline anxiolytic phenotype [10,14], higher firing rates of dorsal raphe 5-hydroxytryptamine or serotonin (5-HT) neurones [15] and also to display treatment resistance to chronic Flx administration [16]. Our results reveal that the anxiogenic effects of acute Flx treatment, as well as the enhanced serum corticosterone levels are unaltered in 5-HT_2A_^−/−^ mice of both sexes. However, 5-HT_2A_ receptor deficiency does alter the pattern of neural activation within the PVN following acute Flx, and the baseline expression of several IEGs in the PFC in a sexually dimorphic manner.

## Experimental procedures

### Animals and drug treatment

Serotonin_2A_ receptor (5-HT_2A_) knockout mice (5-HT_2A_^−/−^) [10] and wild-type (WT) littermate controls of both sexes (4–7 months) were maintained on a 129S6/SvEv background, and group housed on a 12-h normal light–dark cycle, with access to food and water *ad libitum* in the Tata Institute of Fundamental Research (TIFR) animal house facility. Genotypes were confirmed using PCR analysis as described previously [14]. All experimental procedures followed the guidelines of the Committee for Supervision and Care of Experimental Animals (CPCSEA), Government of India, and were approved by the TIFR Institutional Animal Ethics committee in accordance with the National Institutes of Health guide for the care and use of Laboratory animals (NIH Publication No. 8023, revised 1978). The experimental groups were as follows for both sexes: WT + saline (WT Sal), WT + Flx (WT Flx), 5-HT_2A_^−/−^ + saline (5-HT_2A_^−/−^ Sal) and 5-HT_2A_^−/−^ + Flx (5-HT_2A_^−/−^ Flx). Different cohorts were used for each behavioral (open-field test (OFT): males: *n*=10/group; females: *n*=9–10/group; elevated plus-maze test (EPM): males: *n*=11–15/group; females: *n*=12–14/group), cellular (FBJ murine osteosarcoma viral oncogene homolog peptide (c-Fos) profiling) (males: *n*=4–6/group; females: *n*=5–6/group) and molecular (quantitative real-time PCR (qPCR)) (males: *n*=8–12/group; females: *n*=7–10/group) characterization. Serum corticosterone analysis was performed on the same cohort used for cellular analysis (males: *n*=3–6/group; females: *n*=5–6/group). Mice were administered a single intraperitoneal injection of Flx (15 mg/kg, Sigma, U.S.A.) or vehicle (saline), and were subjected to behavioral testing or killed for gene expression analysis 2 h later, or serum corticosterone quantitation and immunohistochemistry analysis after 30 min.

### OFT and EPM

Animals were assessed for anxiety-like response on the OFT as described previously [14]. Briefly, mice were placed in one corner of the arena and allowed to explore the arena for 10 min under dim light conditions. Behavioral tracking was done using Panlab SMART video tracking software (SMART 3.0) and percent distance in center, percent time in center, entries to center and total distances traversed were assessed.

A separate cohort of mice was assessed for anxiety-like behavioral responses on the EPM consisting of adjacent open and closed arms for a duration of 10 min. The mice were placed at the intersection of the four arms facing the open arm, and allowed to explore the arena under dim light conditions. The movement was tracked using an overhead camera and Ethovision 3.1 (Noldus, The Netherlands) video tracking software. The total distance traversed in the EPM arena, percent distance traversed in the open arms, percent time spent in the open arms, and number of entries to the open arms were analyzed.

### Serum corticosterone assay

Blood was collected by cardiac puncture 30 min post a single Flx (15 mg/kg) or saline injection from 5-HT_2A_^−/−^ and WT littermate control mice of both sexes. Serum was collected by transferring the supernatant into a fresh tube and stored at −80°C till further analysis. ELISA was performed to measure serum corticosterone levels using a commercially available ELISA kit (Abcam, Catalog number ab108821) as per the manual (male: *n*=3–6/group; female: *n*=5–6/group).

### c-Fos immunohistochemistry

Animals were perfused with saline, followed by 4% paraformaldehyde (PFA) solution and brains were dissected out and stored in 4% PFA. Brains were sectioned on a vibratome (Leica VT1000 S, U.S.A.) at 40-μm thickness. Free-floating sections spanning the PVN of the hypothalamus (−0.58 to −1.22 mm from Bregma, five to six sections per animal) and PFC (2.46–1.34 mm from Bregma, five to six sections per animal) were subjected to processing for c-Fos immunohistochemistry. Briefly, sections were blocked in 10% horse serum, followed by overnight incubation with rabbit-anti-c-Fos antibody (1:500, Catalog number CST-2250S, Cell Signaling Technology). Sections were washed and incubated with a biotinylated horse-anti-rabbit IgG secondary antibody (1:500, Catalog number BA-1100, Vector Laboratories). Signal amplification and visualization involved exposure to an avidin–biotin complex solution (ABC solution, Vector Laboratories) followed by 3,3′-Diaminobenzidine (DAB) substrate (Sigma, U.S.A.). Sections were mounted and visualized at 200× magnification (Zeiss Axioskop, Germany). c-Fos positive cells were counted per section by an experimenter blinded to the treatment conditions.

### qPCR analysis

Animals were killed 2 h post a single Flx (15 mg/kg) or saline injection by decapitation; the PFC was dissected out and snap-frozen in liquid nitrogen. RNA was extracted using Tri reagent (Sigma) and reverse transcribed using a cDNA synthesis kit (PrimeScript First Strand cDNA Synthesis Kit, Takara Bio). qPCR was performed with primers for the genes of interest (Supplementary Table S1) using a Bio-Rad CFX96 real-time PCR machine. Data were analyzed using the ΔΔ*C*_t_ method, as described previously [17]. Normalization was done using hypoxanthine guanine phosphoribosyl transferase (*Hprt*), whose level was unaltered across experimental groups. Gene expression of IEGs analyzed included activity regulated cytoskeleton associated protein (*Arc*), brain-derived neurotrophic factor (*Bdnf*), early growth response gene 1-4 (*Egr 1-4*), FBJ osteosarcoma gene (*Fos*), FBJ murine osteosarcoma viral oncogene homolog B (*Fosb*), Fos-like antigen 2 (*Fosl2*), Homer scaffolding protein 1-3 (*Homer 1-3*), Jun proto-oncogene (*Jun*), Jun B proto-oncogene (*Junb*), Jun D proto-oncogene (*Jund*). qPCR analysis was also performed for the 5-HT_2A_ gene (*Htr2a*) to further confirm our genotyping results.

### Statistical analysis

Statistical analysis was carried out using Prism 6 (GraphPad Software Inc, U.S.A.). Data were assessed using two-way ANOVA analysis followed by Bonferroni *post-hoc* group comparisons. Normality of data was verified using the Kolmogorov and Smirnov method. Significance was determined at *P*<0.05.

## Results

### 5-HT_2A_^−/−^ mice exhibit acute Flx-induced enhanced anxiety-like behavior in the OFT

5-HT_2A_ receptor knockout (5-HT_2A_^−/−^) mice have been reported to exhibit reduced anxiety-like behavior across both sexes (Supplementary Figure S1) [10,14]. Acute Flx treatment is known to enhance anxiety-like behavioral responses [3]. Here, we sought to address whether the acute Flx-mediated increase in anxiety-like behavior is altered in 5-HT_2A_^−/−^ mice.

**Figure 1:**
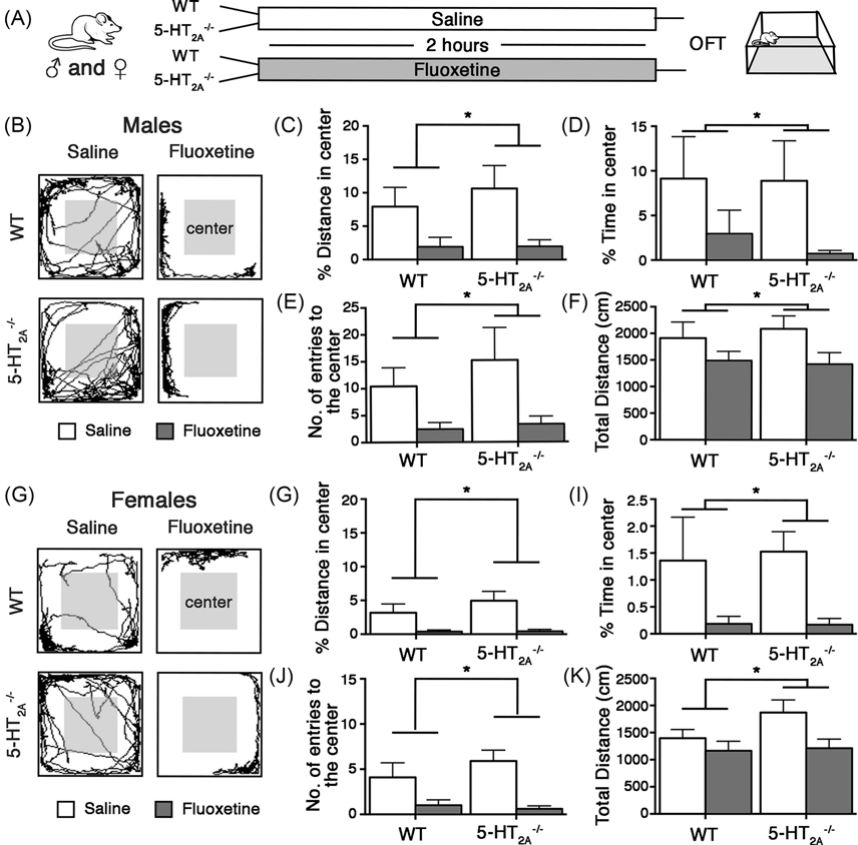
5-HT2A receptor knockout mice exhibit acute Flx-induced enhanced anxiety-like behavior in OFT Shown is a schematic of the experimental paradigm (**A**). 5-HT_2A_^−/−^ and WT littermate control male and female mice were administered acute Flx or saline (Sal), and were subjected to behavioral testing on the OFT 2 h post treatment (A). Shown are representative traces in the OFT arena from saline and Flx treated, WT and 5-HT2A^−/−^ male (**B**) and female (**G**) mice. Both 5-HT2A^−/−^ and WT male (B,**C–F**) and female (G,**H–K**) mice exhibited significant increases in anxiety-like behavior on the OFT following acute Flx treatment, with a reduction noted in percent distance traveled in center (males (C); females (H)), percent time in center (males (D); females (I)) and number of entries to the center (males (E); females (J)) of the OFT arena, as well as the total distance traveled in the OFT arena (males (F); females (K)). Results are expressed as the mean ± S.E.M. (*n*=9–10/group). Two-way ANOVA analysis, *P<0.05, significant main effect of acute Flx treatment.

Male and female 5-HT_2A_^−/−^ and WT littermate control mice were subjected to a single administration of Flx or saline, and were assessed for anxiety-like behavior in the OFT arena for 10 min (Figure 1A). Two-way ANOVA analysis of OFT behavior in male 5-HT_2A_^−/−^ and WT mice revealed a significant main effect of acute Flx administration on percent distance traveled in center (*F*_(1,36)_ = 9.313, *P*=0.0043) (Figure 1C), percent time in center (*F*_(1,36)_ = 4.164, *P*=0.048) (Figure 1D), number of entries to the center (*F*_(1,36)_ = 7.608, *P*=0.0091) (Figure 1E), and the total distance traveled in the OFT arena (*F*_(1,36)_ = 5.17, *P*=0.029) (Figure 1F). Our results indicate a significant effect of acute Flx treatment on anxiety-like behavior in both WT and 5-HT_2A_^−/−^ mice (Figure 1B–F). Interestingly, the baseline decrease in anxiety-like behavior previously noted in 5-HT_2A_^−/−^ mice [10,14], was not observed following administration of a single saline injection, suggesting that the anxiety-like behaviors in 5-HT_2A_^−/−^ mice may be sensitive to both handling and injection stress. There was no significant main effect of genotype and no interaction effect (genotype × Flx).

Similar to our observations in male mice, female 5-HT_2A_^−/−^ and WT littermate controls both showed enhanced anxiety-like behavior on the OFT following acute Flx treatment (Figure 1G–K). Two-way ANOVA analysis of OFT behavior indicated a significant main effect of acute Flx treatment on percent distance traveled in center (*F*_(1,35)_ = 13.54, *P*=0.0008) (Figure 1H), percent time in center (*F*_(1,35)_ = 7.454, *P*=0.0098) (Figure 1I), number of entries to the center (*F*_(1,35)_ = 15.09, *P*=0.0004) (Figure 1J), and total distance traveled (*F*_(1,35)_ = 5.592, *P*=0.0237) in the OFT arena (Figure 1K). We noted no main effect of genotype and no significant interaction (genotype × Flx) effects. This indicates that the baseline anxiolytic responses on OFT reported in both sexes of 5-HT_2A_^−/−^ mice are not observed following handling and injection stress. Taken together, our findings indicate that 5-HT_2A_ receptor deficiency does not influence the increased anxiety-like behavior noted following acute treatment with the SSRI, Flx.

### 5-HT_2A_ receptor knockout mice exhibit acute Flx-induced enhanced anxiety-like behavior in the EPM

**Figure 2:**
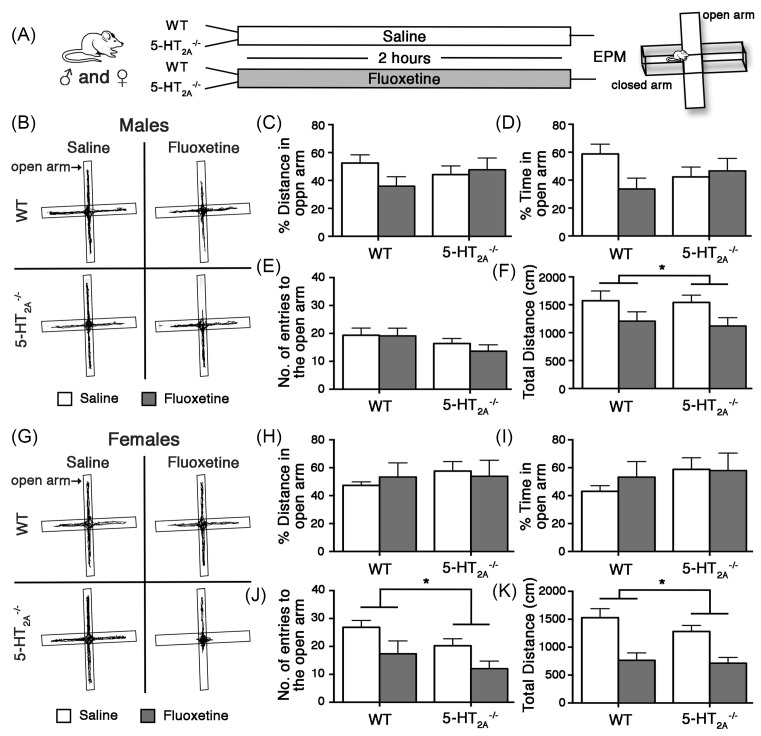
5-HT_2A_^−/−^ mice exhibit acute Flx-induced enhanced anxiety-like behavior on EPM Shown is a schematic of the experimental paradigm (**A**). 5-HT_2A_^−/−^ and WT control male and female mice were administered saline (Sal) or acute Flx, and were subjected to behavioral testing on the EPM 2 h post treatment (A). Shown are representative EPM arena tracks from saline and Flx treated, WT and 5-HT_2A_^−/−^ male (**B**) and female (**G**) mice. Both 5-HT_2A_^−/−^ and WT male (B,**C**–**F**) and female (G,**H**–**K**) mice exhibited significant increases in anxiety-like behavior on the EPM following acute Flx treatment, with a reduction noted in total distance traveled in the EPM arena while percent distance traveled in open arms (males (C); females (H)), percent time in open arms (males (D); females (I)) were unaltered by Flx administration. The number of entries to the open arms of the EPM arena was significantly reduced by Flx in females (J) but not in males (E). Results are expressed as the mean ± S.E.M. (*n*=11–15/group). Two-way ANOVA analysis, *P<0.05, significant main effect of acute Flx treatment.

We next examined the influence of acute Flx on anxiety-like behavior in 5-HT_2A_^−/−^ mice and their WT controls, in the EPM (Figure 2A–K).

Two-way ANOVA analysis of EPM behavior in males revealed no significant main effect of acute Flx on percent distance traveled (Figure 2C), percent time spent (Figure 2D), and number of entries (Figure 2E) in the open arms. For the percent time spent in the open arms for males, we did note a trend toward an interaction of genotype × Flx (*F*_(1,50)_ = 3.39, *P*=0.0714) (Figure 2D), which did not reach statistical significance. Flx treatment significantly reduced the total distance traveled in the EPM arena (*F*_(1,50)_ = 6.42, *P*=0.0145) (Figure 2F), which is considered to be indicative of an enhanced anxiety-like behavioral response. We noted no main effect of genotype and no significant genotype × Flx interaction on any of the EPM parameters in male mice.

Comparable with the male EPM behavioral response, female WT and 5-HT_2A_^−/−^ mice also exhibited enhanced acute Flx-mediated anxiety in the EPM (Figure 2G–K). Two-way ANOVA analysis of EPM in females revealed no significant main effect of acute Flx on percent distance traveled (Figure 2H), and percent time spent (Figure 2I) in the open arms. Acute Flx treatment significantly reduced the number of entries in the open arms (*F*_(1,46)_ = 7.86, *P*=0.0074) (Figure 2J) and the total distance traveled in the EPM arena (*F*_(1,46)_ = 25.3, *P*<0.0001) (Figure 2K), suggestive of enhanced anxiety-like behavior. We noted no main effect of genotype and no significant genotype × Flx interaction for the behavioral measures assayed on the EPM in females.

### 5-HT_2A_ receptor knockout mice exhibit elevated serum corticosterone levels and sexually dimorphic PVN neural activation following acute Flx administration

**Figure 3:**
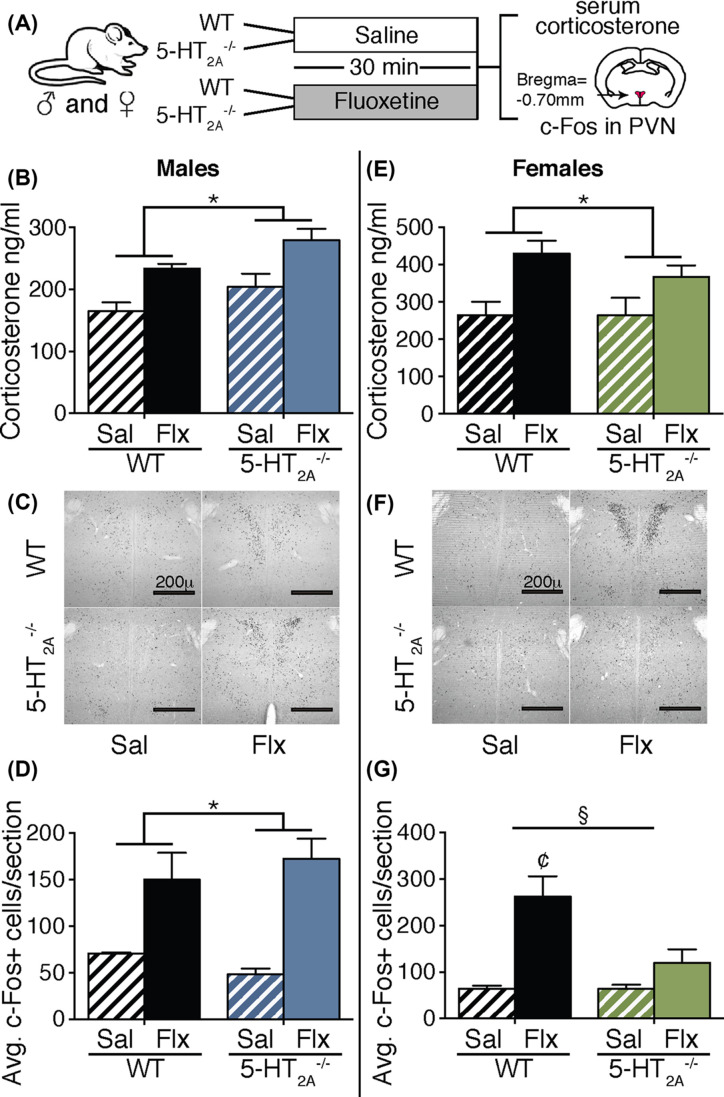
5-HT_2A_ receptor knockout mice exhibit elevated serum corticosterone levels and sexually dimorphic PVN c-Fos positive cell number following acute Flx administration Shown is a schematic of the experimental paradigm (**A**). 5-HT_2A_^−/−^ and WT control male and female mice were administered acute Flx or saline (Sal), and were killed 30 min later for serum corticosterone analysis or immunohistochemical analysis of c-Fos positive cells in the PVN of the hypothalamus (A). Male (**B**) and female (**E**) 5-HT_2A_^−/−^ and WT mice showed an increase in the serum corticosterone levels post Flx treatment. Shown are representative sections of c-Fos positive cells within the PVN of 5-HT_2A_^−/−^ and WT mice treated with saline or Flx (male (C); female (F)). 5-HT_2A_^−/−^ and WT male mice exhibited a significant increase in c-Fos positive cell numbers in the PVN (**D**). A significant genotype × Flx interaction effect was noted for c-Fos positive cell numbers in the PVN of 5-HT_2A_^−/−^ and WT female mice administered Flx or saline (**G**). Post-hoc Bonferroni multiple comparisons revealed a significant increase in PVN c-Fos positive cell numbers following acute Flx in WT, but not in 5-HT_2A_^−/−^, female mice (G). Results are expressed as the mean ± S.E.M. (*n*=3–6/group). Two-way ANOVA analysis, *P<0.05, significant main effect of acute Flx treatment, ^§^*P*<0.05, significant genotype × Flx interaction, Bonferroni post-hoc test, ^¢^*P*<0.05, as compared with saline-treated WT female mice.

Acute Flx treatment is known to increase serum corticosterone levels through activation of the HPA axis [18,19]. Given that the 5-HT_2A_ receptor has been implicated in regulation of HPA axis activity via effects on stimulation of PVN neurones in the hypothalamus [20], we sought to address whether the acute Flx-mediated effects on serum corticosterone and neural activation in the PVN are altered in 5-HT_2A_^−/−^ mice (Figure 3A).

Two-way ANOVA analysis for serum corticosterone levels in males revealed a significant main effect of acute Flx treatment (*F*_(1,15)_ = 15.09, *P*=0.002) and genotype (*F*_(1,15)_ = 5.18, *P*=0.04), but no significance in interaction of genotype and Flx (Figure 3B). We next assessed the number of c-Fos^+^ cells within the PVN of 5-HT_2A_^−/−^ and WT littermate control male mice as a measure of the degree of neural activation following acute Flx treatment. Two-way ANOVA analysis for number of c-Fos positive cells indicated a significant main effect of acute Flx treatment (*F*_(1,18)_ = 24.89, *P*<0.0001) (Figure 3C,D), with no main effect noted for genotype and no genotype × Flx interaction. This indicates that 5-HT_2A_ receptor deficiency in male mice does not alter either the acute Flx-mediated increase in serum corticosterone levels or the increased neural activation, as assessed using c-Fos positive cell numbers, within the PVN.

Similar analysis for serum corticosterone levels carried out in female 5-HT_2A_^−/−^ and WT mice indicated a significant main effect of acute Flx treatment (*F*_(1,17)_ = 13.21, *P*=0.002) with elevated levels of circulating corticosterone noted following acute Flx administration (Figure 3E). No main effect of genotype, or interaction between genotype and Flx treatment, was noted in the two-way ANOVA analysis. The degree of neural activation in the PVN of female 5-HT_2A_^−/−^ and WT mice following acute Flx treatment was assessed using c-Fos cell counting analysis (Figure 3F,G). Two-way ANOVA analysis indicated a significant genotype × Flx interaction (*F*_(1,18)_ = 6.58, *P*=0.02), and significant main effects for acute Flx treatment (*F*_(1,18)_ = 20.57, *P*=0.0003) and genotype (*F*_(1,18)_ = 6.53, *P*=0.02) (Figure 3G). *Post-hoc* group comparisons revealed a significant increase in c-Fos positive cell numbers in the PVN following acute Flx administration to WT, but not 5-HT_2A_^−/−^, female mice (Figure 3G). This suggests that 5-HT_2A_ receptor deficiency in female mice results in a blunting of the degree of neural activation in the PVN following acute Flx administration.

### 5-HT_2A_ receptor deficiency does not alter neural activation patterns in the PFC following acute Flx administration

**Figure 4:**
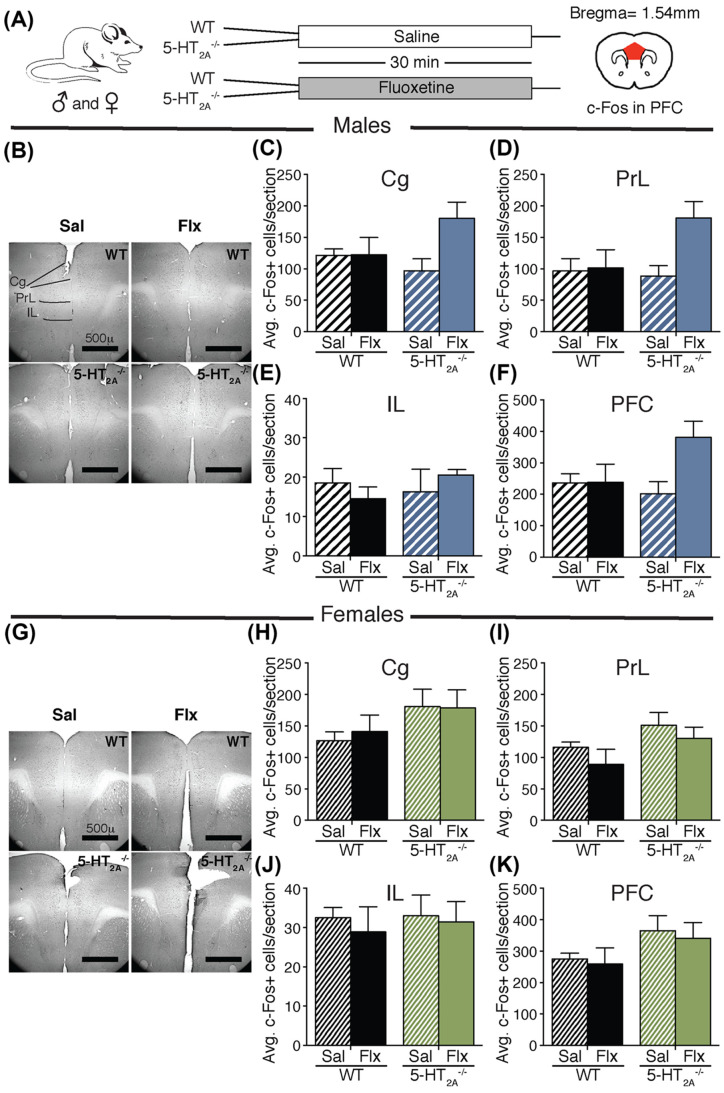
Acute Flx treatment does not alter c-Fos positive cell number in the PFC of 5-HT_2A_^−/−^ and WT male and female mice Shown is a schematic of the experimental paradigm (**A**). 5-HT_2A_^−/−^ and WT control male and female mice were administered acute Flx or saline (Sal), and were killed 30 min later for immunohistochemical analysis of c-Fos positive cell number in the subdivisions of the PFC, namely the Cg, PrL, and IL cortex (A). Shown are representative sections of c-Fos positive cells within the PFC of 5-HT_2A_^−/−^ and WT mice treated with saline or Flx (male (B); female (G)). Cell counting analyses revealed no significant main effect of Flx treatment, genotype or genotype × Flx interaction on c-Fos positive cell numbers within the Cg (male (C); female (H)), PrL (male (D); female (I)), IL (male (E); female (J)) and total PFC (male (F); female (K)). Results are expressed as the mean ± S.E.M. (*n*=5–6/group). Two-way ANOVA analysis.

We next examined neural activation, using c-Fos cell counting analysis, within the subdivisions of the PFC, which are known to exert a strong top-down control on HPA axis activity [21], exhibit robust 5-HT_2A_ receptor expression and are known to regulate anxiety-like behavior [10,22]. We assessed c-Fos positive cell numbers within the cingulate (Cg), prelimbic (PrL) and infralimbic (IL) subdivisions of the PFC in male and female 5-HT_2A_^−/−^ and WT littermate control mice treated with acute Flx or saline (Figure 4A).

Two-way ANOVA analysis for c-Fos positive cell numbers in the Cg, PrL, IL and cumulatively for the entire PFC, in experiments done in 5-HT_2A_^−/−^ and WT male mice indicated no significant main effects of Flx, genotype or genotype × Flx interaction (Figure 4B–F). Similarly, c-Fos positive cell numbers in the Cg, PrL, IL and for the total PFC were unaltered in 5-HT_2A_^−/−^ and WT female mice following acute Flx or saline treatment (Figure 4G–K). Collectively, these findings indicate that neural activation patterns in the PFC subdivisions are not altered in 5-HT_2A_ receptor deficient mice of both sexes.

### 5-HT_2A_ receptor knockout mice exhibit sexually dimorphic alterations in PFC IEG expression

**Figure 5:**
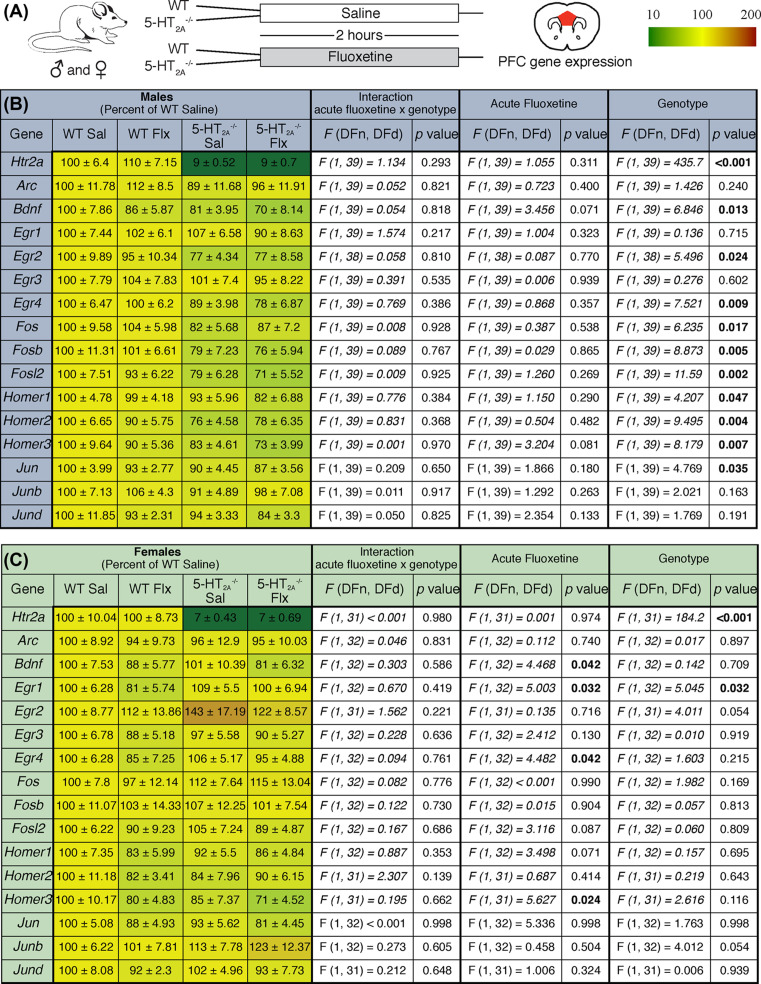
5-HT_2A_ receptor knockout mice exhibit sexually dimorphic changes in PFC IEG expression Shown is a schematic of the experimental paradigm (**A**). 5-HT_2A_^−/−^ and WT control male and female mice were administered acute Flx or saline (Sal), and were killed 2 h post treatment for qPCR analysis of IEG expression in the PFC (A). The heat map denotes the magnitude of regulation represented as percent of WT saline group (WT Sal), with up-regulated genes shown in red and down-regulated genes shown in green (key (A)). qPCR analysis for gene expression of the IEGs examined represented as percent of WT Sal ± S.E.M. (male (B); female (C)) (male: *n*=8–12/group; female: *n*=7–10/group). Two-way ANOVA analysis with significance determined at *P*<0.05. IEGs analyzed included the Arc, Bdnf, Egr 1-4, Fos, Fosb, Fosl2, Homer 1-3, Jun, Junb, and Jund. We also performed qpCR analysis for the *Htr2a* to further confirm our genotyping results.

IEG expression within the neocortex, including the PFC, has been reported to be regulated by 5-HT_2A_ receptors [14,23], hence we sought to address the effects of acute Flx on expression of several IEGs in the PFC of 5-HT_2A_^−/−^ and WT mice of both sexes (Figure 5A).

In the male PFC, two-way ANOVA analysis of qPCR results indicated significant main effects of genotype for several IEGs: *Bdnf, Egr2, Egr4, Fos, Fosb, Fosl2, Homer1-3*, and *Jun* (Figure 5B). No significant main effects of genotype were observed for *Arc, Egr1, Egr3, Junb*, and *Jund* gene expression (Figure 5B). 5-HT_2A_^−/−^ male mice exhibited a decline in PFC gene expression for several IEGs as compared with WT littermate control male mice (Figure 5B). Acute Flx treatment did not alter the mRNA expression levels of the IEGs assessed, except for a strong trend for decline observed in gene expression levels of *Bdnf* and *Homer3* in the male PFC (Figure 5B). We observed no significant interactions of genotype × Flx for gene expression of the IEGs in the PFC in 5-HT_2A_^−/−^ and WT male mice.

In striking contrast with the males, PFC IEG expression was largely unperturbed in the 5-HT_2A_^−/−^ female mice indicating a sexual dimorphism in the effects of the 5-HT_2A_ receptor on PFC IEG expression. Two-way ANOVA analysis indicated no significant main effect of genotype on the gene expression of IEGs in the PFC in females, with the exception of a main effect of genotype observed for *Egr1* gene expression, and a strong trend toward a main effect of genotype in the expression of *Egr2* and *Junb* (Figure 5C). Two-way ANOVA analysis revealed a significant main effect of Flx for *Bdnf, Egr1, Egr4*, and *Homer3* gene expression in the PFC (Figure 5C), and a strong trend for a main effect of Flx in the prefrontal expression of *Fosl2* and *Homer1* (Figure 5C). We observed no significant genotype × Flx interaction for gene expression of the IEGs in the PFC in 5-HT_2A_^−/−^ and WT female mice. Collectively, our results indicate that 5-HT_2A_ receptor deficiency exerts sexually dimorphic consequences on the expression of several IEGs in the PFC, with a decline in PFC expression of *Bdnf, Egr2, Egr4, Fos, Fosb, Fosl2, Homer1-3*, and *Jun* noted in 5-HT_2A_^−/−^ male, but not female mice.

## Discussion

Here, we report that 5-HT_2A_ receptor loss of function does not alter either the acute Flx-mediated increase in anxiety-like behavior, or the enhanced circulating corticosterone, in both male and female mice. However, 5-HT_2A_ receptor knockout mice do exhibit sexually dimorphic effects in the acute Flx-induced neural activation in the PVN with a blunted response to acute Flx noted in 5-HT_2A_^−/−^ female, but not male, mice. Furthermore, we noted sex-dependent changes in expression of several IEGs (*Bdnf, Egr2, Egr4, Fos, Fosb, Fosl2, Homer1-3* and *Jun*) in the PFC of 5-HT_2A_^−/−^ male, but not female, mice.

### 5-HT_2A_ receptor deficiency and acute Flx evoked anxiety-like behavior

The SSRI Flx exerts paradoxical anxiogenic effects during the acute phase of treatment [3,4]. These aversive effects of acute Flx on anxiety-like behavior are suggested to involve a role for the 5-HT_2_ receptor subtypes, with the 5-HT_2C_ receptor in the BNST [6] and BLA [8,12], implicated in these anxiogenic effects. The 5-HT_2A_ receptor also plays an important role in the modulation of anxiety-like behavior [9], and co-administration of a 5-HT_2A_ receptor antagonist is reported to accelerate and augment antidepressant effects of SSRIs [13,24]. Here, we examined whether the increased anxiety-like behavior evoked by acute Flx is altered in 5-HT_2A_^−/−^ male and female mice. Our results indicate that similar to WT mice, adult 5-HT_2A_^−/−^ male and female mice both exhibit acute Flx-induced increases in anxiety-like behavioral responses on the OFT and EPM, indicating that 5-HT_2A_ receptor deficiency does not influence the anxiogenic effects of acute Flx. It would be important in future experiments to address whether 5-HT_2A_ receptor deficient mice which continue to exhibit the acute anxiogenic effects of Flx, also respond in a similar manner to acute treatment with other SSRIs.

Interestingly, while we did observe the previously reported baseline anxiolytic behavioral response in 5-HT_2A_^−/−^ male and female mice (Supplementary Figure S1) [10,14], the vehicle-treated 5-HT_2A_^−/−^ cohorts used for the acute Flx experiment did not exhibit this baseline anxiolytic phenotype. The cohorts used for the present study were subjected to handling and injection of vehicle/Flx, and since the anxiolytic behavioral phenotype observed in naïve, unhandled 5-HT_2A_^−/−^ mice was lost, this suggests the possibility that 5-HT_2A_ receptor deficient mice may be highly sensitive to effects of handling and injection stress. Prior reports do indicate that handling and injection stress can alter anxiety-like behaviors [25,26]. Further experiments are required to directly test whether 5-HT_2A_ receptor deficient mice exhibit such enhanced sensitivity to handling and injection stress.

A previous report indicates that 5-HT_2A_^−/−^ mice exhibit treatment resistance to chronic Flx, failing to exhibit both antidepressant-like behavioral responses and enhanced hippocampal neurogenesis following chronic Flx treatment [16]. Taken together, these findings indicate that while the anxiogenic effects noted during the acute phase of Flx treatment are unaltered by 5-HT_2A_ receptor deficiency, 5-HT_2A_^−/−^ mice exhibit a treatment-resistant phenotype to the effects of chronic Flx [16].

### Role of the 5-HT_2A_ receptor in the effects of acute Flx on the HPA axis

Acute Flx treatment is known to influence the HPA axis, increasing circulating levels of corticosterone via enhanced activity of PVN neurones in the hypothalamus [18,19]. 5-HT_2A_ receptors are densely expressed by PVN neurones, and their activation leads to release of adrenocorticotrophic hormone and enhanced circulating corticosterone [20,27]. We find that the acute Flx-mediated elevation of serum corticosterone levels is observed in both WT and 5-HT_2A_^−/−^ male and female mice, indicating that the effects of acute Flx on enhanced corticosterone levels do not appear to involve a role for the 5-HT_2A_ receptor.

Acute Flx administration also resulted in a clear increase in neural activation within the PVN, as revealed by enhanced numbers of c-Fos positive cells. This acute Flx-evoked increase in PVN c-Fos positive cells was noted in both WT and 5-HT_2A_^−/−^ male mice, and the magnitude of this effect was unaltered by 5-HT_2A_ receptor deficiency in males. Strikingly, 5-HT_2A_^−/−^ female mice showed a significant blunting of the acute Flx-mediated increase in c-Fos positive cells in the PVN. Previous studies indicate an interaction of sex hormones such as estrogen with SSRIs such as Flx [28,29], and also with the 5-HT_2A_ receptor [30,31]. This in turn provides support for the possibility that the effects of acute Flx on neural activation in the PVN may exhibit sex differences, possibly through interactions between the 5-HT_2A_ receptor, estrogen and acute Flx treatment. While we noted that the acute Flx-mediated increase in c-Fos positive cell number in the PVN was blunted in 5-HT_2A_^−/−^ female mice, we did not observe any significant genotype × Flx interaction for corticosterone levels in 5-HT_2A_^−/−^ female mice. This suggests that despite a substantial blunting of the neural activation in the PVN evoked by acute Flx in 5-HT_2A_^−/−^ female mice, this does not translate to a failure of acute Flx to increase circulating corticosterone in 5-HT_2A_^−/−^ female mice. Collectively, our findings indicate that the effects of acute Flx on enhancing corticosterone levels are unaltered by 5-HT_2A_ receptor deficiency, however 5-HT_2A_ receptor loss of function does alter the degree of neural activation induced by acute Flx in the PVN in a sex-dependent manner.

### 5-H_T2A_ receptor and the acute Flx-evoked regulation of neural activation, and IEG expression, in the PFC

Acute Flx treatment is known to rapidly elevate serotonin levels within the PFC [32], a limbic brain region that exerts top-down control over the HPA axis and regulates anxiety behaviors [21]. The 5-HT_2A_ receptor is expressed at particularly high levels within the PFC, and the cortical 5-HT_2A_ receptor plays a key role in the modulation of anxiety behavior [10,22,33]. We sought to address whether 5-HT_2A_ receptor deficiency altered neural activation measured via assessing c-Fos positive cell number, or activity-dependent IEG expression, in the PFC, both under baseline conditions and in response to acute Flx administration. We observed no difference in c-Fos positive cell numbers in the PFC across groups 30 min post acute Flx administration. Our results do not preclude the possibility of differences in the nature and degree of neural activation of the PFC in WT and 5-HT_2A_^−/−^ mice at later time-points post Flx treatment.

We also examined the expression of several IEGs in WT and 5-HT_2A_^−/−^ male and female mice, both baseline and post Flx treatment. While we did not observe any major effect of Flx treatment on IEG expression in the PFC, we noted robust baseline down-regulation of several IEGs (*Bdnf, Egr2, Egr4, Fos, Fosb, Fosl2, Homer1-3, Jun*) in the PFC of 5-HT_2A_^−/−^ male, but not female mice. Our results are largely in agreement with prior reports that acute Flx, unlike chronic treatment, does not appear to alter cortical IEG and plasticity-associated gene expression [34]. We have previously reported a sex-dependent, baseline regulation of several IEG and plasticity-associated genes in both the PFC and hippocampus of 5-HT_2A_^−/−^ mice [14]. This decline in basal expression of several IEGs in the PFC of 5-HT_2A_^−/−^ male mice may arise as a consequence of reduced excitability of PFC pyramidal neurones following 5-HT_2A_ receptor deficiency, however the fact that this change is not observed in 5-HT_2A_^−/−^ female mice is intriguing. Prior reports suggest an interaction between estrogen and the 5-HT_2A_ receptor with regard to the transcriptional regulation of *Bdnf* [31]. In particular, *Bdnf* expression is reported to be regulated by estrogen receptors via an estrogen response element [35], it is possible that such a regulation may contribute to the differential effects of 5-HT_2A_ receptor deficiency on cortical *Bdnf* expression in male compared with female mice [36]. Our findings suggest a sexual dimorphism in the 5-HT_2A_ receptor-mediated regulation of activity-dependent IEG expression, and motivate future experiments to address the nature of interaction between sex hormones and the 5-HT_2A_ receptor in this regard.

## Conclusion

The findings of our study indicate that the 5-HT_2A_ receptor does not appear to play a major role in the effects of acute Flx on anxiety-like behavior and corticosterone secretion. However, 5-HT_2A_ receptor deficiency evokes sex-dependent differences in both baseline cortical IEG expression, and in the acute Flx-mediated neural activation of the PVN of the hypothalamus. These findings highlight the sex differences in the effects of the 5-HT_2A_ receptor in the regulation of several genes, implicated in neuronal plasticity, within key brain regions such as the PFC. This suggests the possibility of an important interaction between the 5-HT_2A_ receptor, estrogen and activity-dependent IEG and plasticity-associated gene expression, motivating future experiments to uncover the mechanistic details of such an interaction.

## Supplementary Material

Supplementary Figures

Supplementary Table

## Abbreviations

5-HT_2A_: serotonin_2A_ receptor
5-HT_2A_^−/−^: 5-HT_2A_ developmental knockout
5-HT_2C_: serotonin_2C_ receptor
*Arc*: activity regulated cytoskeletal associated protein
Avg: average
*Bdnf*/BDNF: brain derived neurotrophic factor
BLA: basolateral nucleus of the amygdala
BNST: bed nucleus of the stria terminalis
c-Fos: FBJ murine osteosarcoma viral oncogene homolog peptide
Cg: cingulate cortex
Flx: fluoxetine
*Fos*: FBJ osteosarcoma oncogene
*Fosb*: FBJ murine osteosarcoma viral oncogene homolog B
*Fosl2*: Fos-like antigen 2
*Homer*: Homer scaffolding protein
HPA axis: hypothalamus–pituitary–adrenocortical axis
IEG: immediate early gene
IL: infralimbic cortex
*Jun*: Jun proto-oncogene
OFT: open-field test
PFA: paraformaldehyde
PFC: prefrontal cortex
PrL: prelimbic cortex
PVN: paraventricular nucleus
qPCR: quantitative real-time PCR
SSRI: selective serotonin reuptake inhibitor
WT: wild-type
