# Supplementary material for: 5-HT2A receptor loss does not alter acute fluoxetine-induced anxiety and exhibit sex-dependent regulation of cortical immediate early gene expression
Source: Neuronal Signal. 2019 Feb 1;3(1):NS20180205. doi: 10.1042/NS20180205 (PMC7363295; doi:10.1042/NS20180205)
Supplement: Supplementary Figures [file NS-2018-0205_supp.pdf]

Supplementary Material

1. Methods

1.1 Animals

Male and female, 4-7 month-old, wild-type (WT) and serotonin2A receptor knockout (5-$HT_{2A}^{-/-}$) littermate mice on 129S6/SvEv were group-housed under normal 12-hour light-dark cycle with food and water *ad libitum*. The animals were bred and housed in Tata Institute of Fundamental Research animal facility. Three different cohorts of animals were tested for depressive-like and anxiety-like behaviors under baseline condition as mentioned below.

Cohort 1: Tail suspension test and Forced swim test *n* = 14-21/group

Cohort 2: 10-min Open-field test *n* = 6-9/group

Cohort 3: 10-min Elevated-plus maze test *n* = 12-15/group

1.2 Tail suspension test (TST) and Forced swim test (FST)

Male and female, control wild-type (WT) and serotonin2A receptor knockout (5-$HT_{2A}^{-/-}$) were subjected to five minutes of tail suspension test (TST), and two-days later to five minutes of Porsolt's classic forced-swim test (FST) as previously described (Castagné et al., 2011). The videos were recorded using a side camera and analyzed manually by an experimenter blind to the treatment conditions or the genotype. The immobility time was manually determined from the first to the fifth minute and time spent immobile in seconds in represented.

1.2 Statistical Analysis

Statistical analysis was carried out using Prism 6 (GraphPad Software Inc, USA). The normality of the data was tested using Kolmogorov and Smirnov method. Two-way ANOVA with Bonferroni *post-hoc* test was carried out for all behavioral

experiments and significance was determined at $p<0.05$. Data are represented as mean ± SEM.

2. Results:

2.1 5-$HT_{2A}^{-/-}$ mice are comparable to WT on despair-like behavior, but show reduced anxiety-like behavior on OFT but not EPM task.

We analyzed three-different cohorts of male and female, WT and 5-$HT_{2A}^{-/-}$ on TST and FST, OFT, or EPM tasks (Fig. S1A). Our data on time spent immobile in TST and FST (Fig. S1B and S1C) revealed no significant effect of genotype or sex. We further assessed a separate cohort of male and female, WT and 5-$HT_{2A}^{-/-}$ mice on the open-field test for a duration of ten minutes. In this task, we observed a strong two-way ANOVA genotype effect for the following measures: total distance ($F_{(1,28)}$ = 13.70, p = 0.0009) (Fig. S1D), number of entries to the center ($F_{(1,28)}$ = 9.098, p = 0.005) (Fig. S1E), percent distance in center ($F_{(1,28)}$ = 13.57, p = 0.001) (Fig. S1F) and percent time pent in center ($F_{(1,28)}$ = 7.103, p = 0.0126) (Fig. S1G). These measures on the OFT revealed significant anxiolytic effects in 5-$HT_{2A}^{-/-}$ mice. There was no interaction (genotype x sex) or main effect of sex observed in any of the OFT measures.

We next analyzed a separate cohort of WT and 5-$HT_{2A}^{-/-}$ mice on the EPM behavioral task for a duration of ten minutes. Similar to OFT, two-way ANOVA analysis revealed that the 5-$HT_{2A}^{-/-}$ mice exhibited reduced anxiety-like behavior in the EPM arena as indicated by increased total distance travelled in the EPM arena ($F_{(1,51)}$ = 4.39, p = 0.0412) (Fig. S1 H) and percent distance travelled in the open arms ($F_{(1,51)}$ = 4.53, p = 0.0382) (Fig. S1 J). We also noted a strong trend towards increased percent time spent in the open arms ($F_{(1,51)}$ = 3.85, p = 0.0551) (Fig. S1 K) in the 5-$HT_{2A}^{-/-}$ mice compared to their WT controls. We did not observe a main effect of genotype in the number of entries to the open arms of the EPM arena (Fig. S1 I). There was no genotype x sex interaction in the two-way ANOVA analysis in any of the EPM measures, however a main effect of sex was noted in the percent time spent in the open arms ($F_{(1,51)}$ = 6.93, p = 0.0112) (Fig. S1 K).

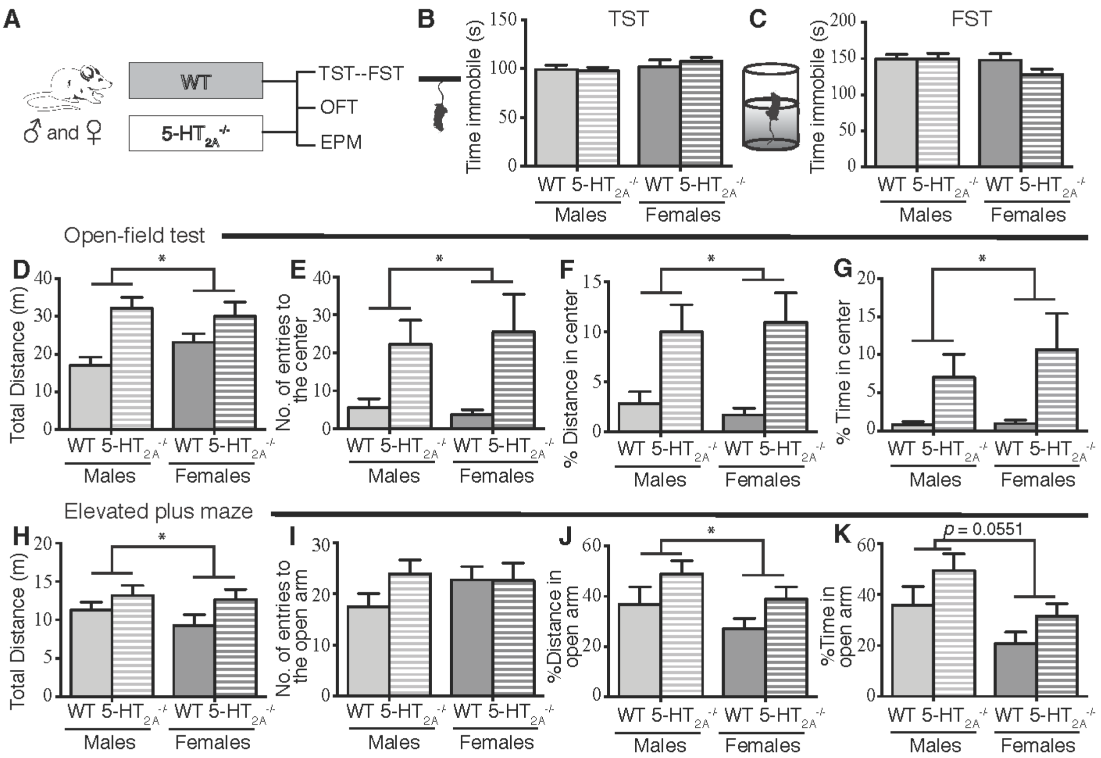

Fig. S1 *Despair-like and anxiety-like behavioral analysis of WT and 5-HT$_{2A}^{-/-}$ mice under baseline conditions.* Shown is a schematic of the experimental paradigm, (A) with three different cohorts used for tail suspension test followed by forced-swim test (TST-FST), open-field test (OFT), and elevated-plus maze test (EPM). Time spent immobile in seconds in TST (B). Time spent immobile in seconds in FST (C). Male and female 5-HT$_{2A}^{-/-}$ mice exhibited a significant reduction in anxiety-like behavior on the OFT task, with an increase noted in total distance travelled in the OFT arena (D), number of entries to the center (E), percent distance travelled in center (F), and percent time spent in the center (G) of the OFT arena. Male and female 5-HT$_{2A}^{-/-}$ mice exhibited a significant reduction in anxiety-like behavior on the EPM task, with an increased total distance travelled in the EPM arena (H), no change in number of entries to the open arms (I), increased percent distance travelled in the open arms (J), and percent time spent in the open arms (K) of the EPM arena. There was no interaction or main effect of sex observed on any of the measures of TST, FST, and OFT. There was no genotype x sex interaction effect on any of the EPM measures and a main effect of sex was observed only for time spent in open arms (K) of the EPM arena. Data represented at Mean ± SEM. Results are expressed as the mean ± SEM. Two-way ANOVA analysis, *$P < 0.05$, significant main effect of genotype.

Reference:

Castagné, V., Moser, P., Roux, S., and Porsolt, R.D. (2011). Rodent Models of Depression: Forced Swim and Tail Suspension Behavioral Despair Tests in Rats and Mice. In Current Protocols in Neuroscience, (Hoboken, NJ, USA: John Wiley & Sons, Inc.), p. Unit 8.10A.
